# Identification of *MAMDC1* as a Candidate Susceptibility Gene for Systemic Lupus Erythematosus (SLE)

**DOI:** 10.1371/journal.pone.0008037

**Published:** 2009-12-07

**Authors:** Anna Hellquist, Marco Zucchelli, Cecilia M. Lindgren, Ulpu Saarialho-Kere, Tiina M. Järvinen, Sari Koskenmies, Heikki Julkunen, Päivi Onkamo, Tiina Skoog, Jaana Panelius, Anne Räisänen-Sokolowski, Taina Hasan, Elisabeth Widen, Iva Gunnarson, Elisabet Svenungsson, Leonid Padyukov, Ghazaleh Assadi, Linda Berglind, Ville-Veikko Mäkelä, Katja Kivinen, Andrew Wong, Deborah S. Cunningham Graham, Timothy J. Vyse, Mauro D'Amato, Juha Kere

**Affiliations:** 1 Department of Biosciences and Nutrition, Karolinska Institutet, Huddinge, Sweden; 2 Wellcome Trust Centre for Human Genetics, University of Oxford, Oxford, United Kingdom; 3 Oxford Centre for Diabetes, Endocrinology and Medicine, University of Oxford, Oxford, United Kingdom; 4 Department of Dermatology, University of Helsinki, and Skin and Allergy Hospital, Helsinki, Finland; 5 Department of Clinical Science and Education and Section of Dermatology, Karolinska Institutet at Stockholm Soder Hospital, Stockholm, Sweden; 6 Department of Medical Genetics, University of Helsinki, and Folkhälsan Institute of Genetics, Helsinki, Finland; 7 Department of Medicine, Helsinki University Hospital and Peijas Hospital, Vantaa, Finland; 8 Department of Biological and Environmental Sciences, University of Helsinki, Helsinki, Finland; 9 Department of Pathology and Transplantation Laboratory, Helsinki University Central Hospital, Helsinki, Finland; 10 Department of Dermatology, Tampere University Hospital and University of Tampere, Tampere, Finland; 11 Department of Medicine, Rheumatology Unit, Karolinska Institutet/Karolinska University Hospital, Stockholm, Sweden; 12 Clinical Research Centre, Karolinska University Hospital, Huddinge, Sweden; 13 The Wellcome Trust Sanger Institute, Wellcome Trust Genome Campus, Hinxton, Cambridge, United Kingdom; 14 Rheumatology Section, Imperial College, Hammersmith Hospital, London, United Kingdom; 15 Imperial College, Molecular Genetics and Rheumatology Section, Hammersmith Hospital, London, United Kingdom; University of Missouri-Kansas City, United States of America

## Abstract

**Background:**

Systemic lupus erythematosus (SLE) is a complex autoimmune disorder with multiple susceptibility genes. We have previously reported suggestive linkage to the chromosomal region 14q21-q23 in Finnish SLE families.

**Principal Findings:**

Genetic fine mapping of this region in the same family material, together with a large collection of parent affected trios from UK and two independent case-control cohorts from Finland and Sweden, indicated that a novel uncharacterized gene, *MAMDC1* (MAM domain containing glycosylphosphatidylinositol anchor 2, also known as MDGA2, MIM 611128), represents a putative susceptibility gene for SLE. In a combined analysis of the whole dataset, significant evidence of association was detected for the *MAMDC1* intronic single nucleotide polymorphisms (SNP) rs961616 (*P* –value = 0.001, Odds Ratio (OR) = 1.292, 95% CI 1.103–1.513) and rs2297926 (*P* –value = 0.003, OR = 1.349, 95% CI 1.109–1.640). By Northern blot, real-time PCR (qRT-PCR) and immunohistochemical (IHC) analyses, we show that *MAMDC1* is expressed in several tissues and cell types, and that the corresponding mRNA is up-regulated by the pro-inflammatory cytokines tumour necrosis factor alpha (TNF-α) and interferon gamma (IFN-γ) in THP-1 monocytes. Based on its homology to known proteins with similar structure, MAMDC1 appears to be a novel member of the adhesion molecules of the immunoglobulin superfamily (IgCAM), which is involved in cell adhesion, migration, and recruitment to inflammatory sites. Remarkably, some IgCAMs have been shown to interact with ITGAM, the product of another SLE susceptibility gene recently discovered in two independent genome wide association (GWA) scans.

**Significance:**

Further studies focused on MAMDC1 and other molecules involved in these pathways might thus provide new insight into the pathogenesis of SLE.

## Introduction

SLE (MIM152700) is a multisystemic autoimmune disorder, with varying incidence and prevalence between populations [1]. The disease is characterized by autoantibody production against self, formation of immune complexes, and subsequent tissue inflammation in multiple organs such as the skin, joints, kidneys and heart. Although the underlying pathogenic mechanisms of SLE remain imperfectly understood, both environmental influences and genetic factors have been found to play an important role in disease initiation and progression [2]–[4]. Supporting a genetic component in SLE, genome-wide linkage scans have identified several loci showing significant linkage to the disease, some of which have been confirmed in independent studies (reviewed in [5]–[10]). In particular, the importance of the two chromosomal regions 6p22.3–p21.1 (HLA region) and 16p12.3–q12.2 has been highlighted in a meta-analysis of genome wide linkage studies in SLE [11]. In addition to these loci, a large number of genes and genetic effects have been associated to SLE through candidate gene studies (reviewed in [5]–[9]). Recent GWA studies have further provided new fundamental insight into the genetics of SLE by identifying new susceptibility genes and consolidating results obtained in previous studies [12]–[15].

Our group previously reported suggestive linkage on chromosomes 5p (Nonparametric linkage (NPL) score = 2.03, *P*-value = 0.02), 6q25-q27 (NPL score = 2.47, *P*-value = 0.008), 14q21-q23 (NPL score = 2.20, *P*-value = 0.02) as well as the HLA region (NPL score = 2.17, *P*-value = 0.02) in a genome-wide scan of 35 Finnish multiplex families [16]. Following up the loci on chromosome 6q and 14q, with an additional 31 Finnish simplex families included in the cohort, we identified sharing of two common haplotypes on chromosome 14q21-23 (spanning markers D14S978-D14S589-D14S562 and D14S1009-D14S748, *P*-value = 0.006 and 0.14 respectively), and excess transmission of a haplotype (GATA184A08-D6S1637, *P*-value = 0.07) on 6q [17]. The chromosome 14 region had previously been reported as a suggestive SLE susceptibility locus in two other independent studies [18], [19] as well as in Systemic Sclerosis (MIM 181750) [20], and was thus subsequently considered of particular interest for gene identification.

In the present study, we have taken a hierarchical, multistep approach to delineate the SLE susceptibility locus contained within the chromosome 14q21-14q23 region and identified a gene, *MAMDC1*, as a novel candidate gene for SLE.

## Results

**Figure 1:**
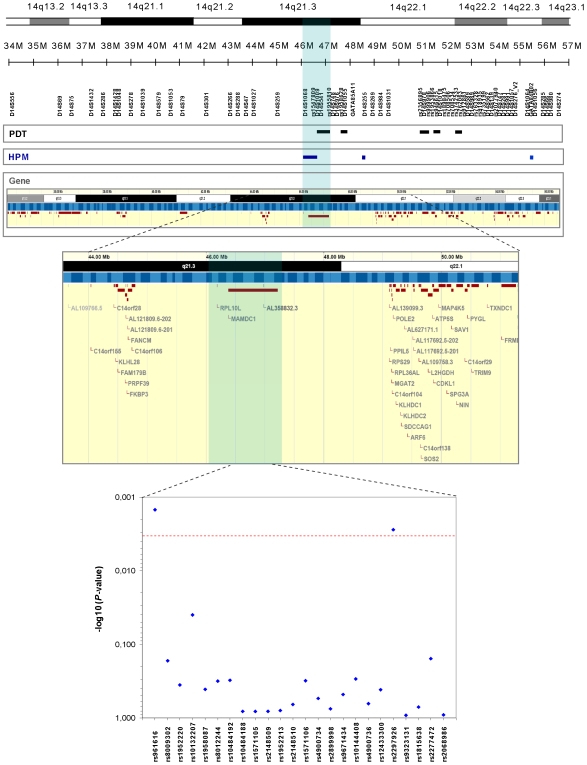
Identification of *MAMDC1* as the SLE susceptibility locus in the 14q21-q23 linkage region. Fine mapping towards the identification of *MAMDC1* was first performed in the original Finnish family cohort by genotyping 19 MS markers and 17 SNPs, focusing on the two regions located on 14q11.2-q23 that showed haplotype sharing in the previous fine-mapping [16], [17]. PDT and HPM analyses were used and regions providing significant results (p≤0.05) are shown in black and blue, respectively (top of the figure). The region between markers D14S1068 and rs1955810, containing the gene *MAMDC1*, was selected for further fine mapping (highlighted in blue). Twenty-four SNPs were subsequently genotyped in the whole sample material and as shown graphically in the figure, significant association for the SNPs rs961616 and rs2297926 could be identified using a combined analysis (bottom of the figure). The significance threshold were set to *P* = 0.0032 (see the statistics section under material and methods) and is represented by a red line.

The original Finnish cohort, with an additional 126 families, was used in the initial fine mapping, which focused on the two regions showing haplotype sharing in the previous fine-mapping [16], [17]. This step of genotyping included 19 microsatellite (MS) markers and 17 SNPs, located within a region spanning 14q11.2-q23 (Figure 1 and Table S1). Together with the MS markers already analyzed in our previous studies [16], [17] genotyping data were thus available in this initial step for 47 MS markers and 17 SNPs, corresponding to an average marker distance of 350 kilo bases (kb) in the region. To increase the chances of identifying SLE susceptibility loci in this region, two different statistical analyses were performed on genotyped data; Pedigree Disequilibrium Test (PDT) [21] and Haplotype Pattern Mining (HPM) [22]. As graphically reported in Figure 1, only one region provided positive signals of association with both methods, namely the 800 kb sequence contained between markers D14S1068 and rs1955810. The poorly characterized gene *MAMDC1* maps right in the middle of and are entirely contained within this region. We therefore focused our downstream analysis onto this locus.

To further explore the observed association, three additional SLE cohorts were included in the study; one family cohort from the UK consisting of 365 SLE parent affected trios, one case control cohort from Finland consisting of 86 SLE cases and 356 controls and one case control cohort from Sweden consisting of 304 SLE cases and 307 controls (see Material and methods and Table S1). Twenty four SNPs, spanning the *MAMDC1* gene, were thus subsequently genotyped in all four sample populations to obtain information regarding the genetic contribution of *MAMDC1* in the European population (Figure 1 and Table S1). A combined analysis, including all four populations, revealed that two SNPs at the *MAMDC1* locus; rs961616 (*P* –value = 0.001, OR = 1.292, 95% CI 1.103–1.513) and rs2297926 (*P* – value = 0.003, OR = 1.349, 95% CI 1.109–1.640) significantly contribute to SLE susceptibility after correction for multiple testing. A graphical view of the *P* – value distribution of the 24 markers is shown in Figure 1 and exact values are shown in Table S2. Haplotype analysis was also performed for each sample population but this did not add any further information.

**Figure 2:**
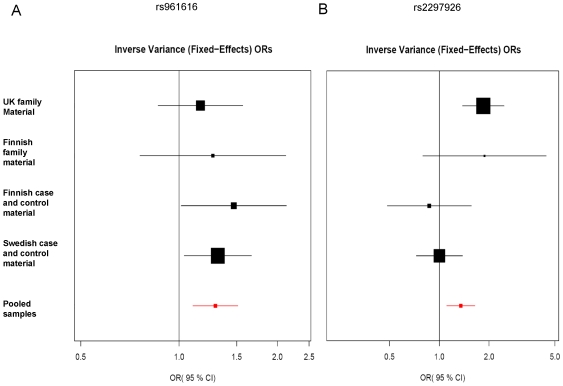
Individual and pooled odds ratios for rs961616 and rs2297926. The individual and pooled contribution of each sample population for rs961616 (A) and rs2297926 (B), shown as ORs.

Despite some individual differences between the study populations, no significant findings were obtained by heterogeneity testing, and the pooled ORs for rs961616 and rs2297926 both remained above the threshold of significance (Figure 2), thus suggesting *MAMDC1* as a candidate gene in SLE susceptibility.

**Figure 3:**
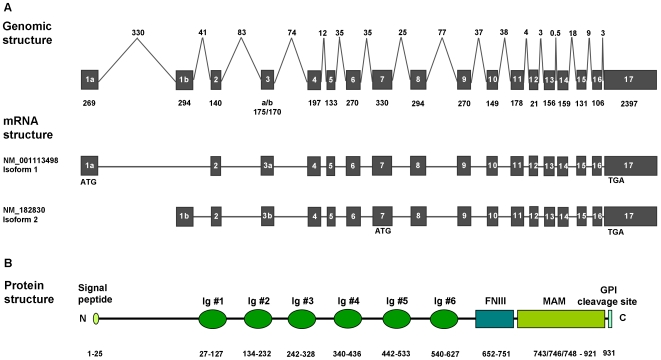
Human MAMDC1 gene, mRNA and protein structure. A) *MAMDC1* genomic structure and exon-intron organization. Exons are reported as plain boxes with relative length (in bp) below. Intronic intervening sequences are also shown with relative length (in kb) above. Two alternative *MAMDC1* mRNA isoforms are predicted to be transcribed from the MAMDC1 locus, corresponding to NCBI database entries NM_001113498 and NM_182830. Translation initiation codons (ATG) are indicated for both isoforms. B) Schematic representation of MAMDC1 predicted full-length protein (corresponding to mRNA isoform 1), and its structural domains with relative length (amino acid positions) below.

**Figure 4:**
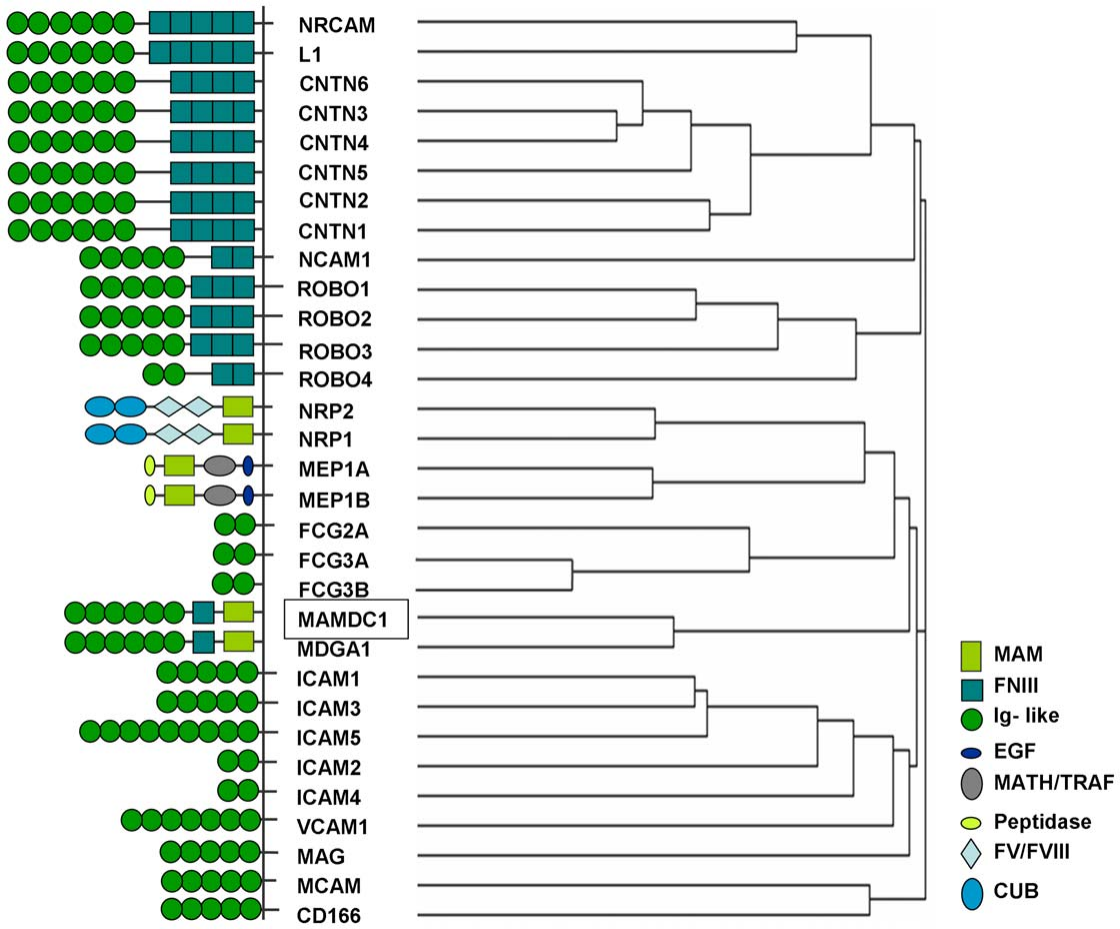
Comparison of homologs of the human MAMDC1 protein. Alignment of MAMDC1 full-length polypeptide with other proteins containing identical structural domains (reported on the left: MAM, meprin/A5-protein/PTPmu; FNIII, fibronectin, type III-like fold, Ig-like, immunoglobulin-like; EGF, epidermal growth factor; MATH/TRAF, meprin and TRAF-C homology/TNF-receptor associated factor; FV/FVIII, coagulation factor V/VIII; CUB, complement C1r/C1s, Uegf, Bmp1). The branching diagram (cladogram) was generated by multiple sequence alignment of the protein sequences using ClustalW [60].

*MAMDC1* is a poorly characterized gene spanning 835 kb of DNA sequence on the reverse strand of Chromosome 14q21.3, and predicted to be composed of two alternative first exons (1a and 1b) and 16 downstream exons giving rise to two mRNAs of similar size (5375 bp for isoform 1, and 5239 bp for isoform 2, respectively; Figure 3A). These correspond to MAMDC1 full-length protein (956 amino acids [aa], Figure 3B) and a shorter peptide where translation is predicted to start at an internal ATG codon from exon 7 (727 aa, not shown). Strikingly, the former shows >97% aa identity with chimpanzee, mouse, rat, dog and horse MAMDC1 orthologs, suggesting an important function for this highly conserved protein. As shown in Figure 3B, analysis of human MAMDC1 full length amino acid sequence with the protein prediction tool InterPro Scan [23] revealed the presence of 6 immunoglobulin (Ig) like domains, a fibronectin type III like (FNIII) fold domain, and a meprin/A5-protein/PTPmu (MAM) domain in the corresponding polypeptide chain. In addition, a C-terminal GPI anchoring signal was detected using big-PI Predictor [24], and an N-terminal signal sequence with SignalP [25]. Interestingly, identical domain structures are found in the human homolog MDGA1 (MIM 609626) [26], [27] and in the rat orthologs MDGA1 and MDGA2 previously identified in different neuronal populations [28]. Based on their protein architecture and pattern of expression, MDGA proteins have been proposed as a novel subgroup of the IgCAM super family, an important class of membrane proteins involved in cell-cell adhesion, migration and the development of neuronal connections [29]. An alignment of MAMDC1 with these and other proteins sharing similar domains and suggested to have a role in adhesion is reported in Figure 4.

**Figure 5:**
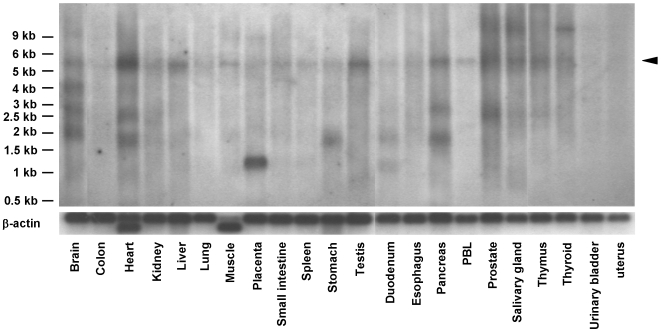
Analysis of MAMDC1 mRNA expression. Northern blot analysis of MAMDC1 mRNA expression in different human tissues, showing several expressed splice variants. *MAMDC1* mRNA transcript corresponding to the full-length isoform is indicated by an arrowhead on the right side. A β-Actin cDNA control probe was used for normalization (bottom).

In order to initially characterize *MAMDC1* distribution in different tissues and cells, we sought to analyze its mRNA and protein expression patterns, respectively by Northern blot hybridization and IHC experiments. As shown in Figure 5, a weak signal corresponding to the expected *MAMDC1* mRNA size of 5 kb was seen in all tissues except urinary bladder and uterus after hybridization of the full-length cDNA probe (corresponding to MAMDC1 isoform 1) on two human multiple tissue polyA+ RNA Northern blots. Several additional transcripts of smaller size were also present in tissues such as the brain, heart, pancreas and others, while a band of approximately 9 kb was further seen in brain, placenta and thyroid, thus suggesting that *MAMDC1* primary transcript undergoes alternative splicing. Hence, our results indicate that human *MAMDC1* has a much broader expression than its MDGA2 rat ortholog [28], as it is found at a low level in a wide range of tissues outside the nervous system.

**Figure 6:**
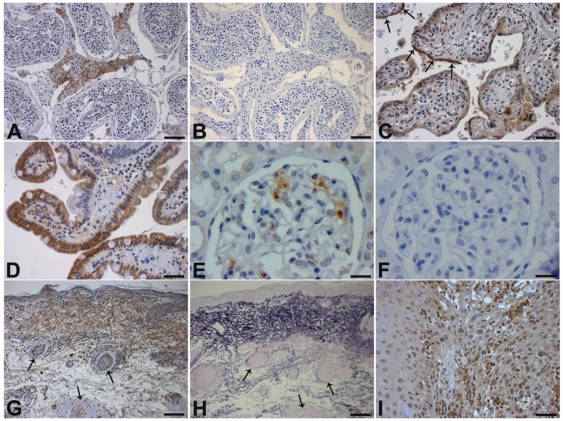
Analysis of MAMDC1 protein expression. IHC analysis of MAMDC1 protein expression in A) testis, showing positive staining in Leydig cells; B) testis, negative control; C) placenta, showing positive staining in syncytial trophoblasts (arrows); D) positive duodenal villi; E) kidney, positive staining observed in occasional glomerular neutrophils; F) kidney, negative control; G) SLE skin with positive staining in the upper dermis in the same region as elastic fibres; H) SLE skin stained with Weigert's Resorcin-Fuchsin, detecting elastic fibers: arrows in G and H mark corresponding regions I) Cutaneous squamous cell cancer, with positive staining in neutrophils. Scale bars: 5 µm (A, B, G, H), 2.5 µm (C, D, I), 1.6 µm (E, F).

To confirm and extend these results, we then studied MAMDC1 protein expression by IHC on formalin-fixed paraffin-embedded tissue sections from testis, kidney, duodenum, placenta, cutaneous squamous cell carcinoma, and SLE skin. A rabbit anti-MAMDC1 antibody was used for this purpose and as shown in Figure 6, MAMDC1 protein expression could be detected in Leydig cells of the testis, in placental syncytial trophoblasts and epithelial cells of the duodenal villi (Figures 6A, 6C and 6D respectively). Further, both kidney and cutaneous squamous cell carcinomas showed positive staining in neutrophils (Figures 6E and 6I). In skin samples obtained from SLE patients the protein was detected in elastic fibres in the upper layers of dermis (Figures 6G and 6H). The results obtained with IHC are in accordance with the data obtained from the analysis of mRNA expression, and further support the finding that MAMDC1 is expressed in tissues other than the nervous system.

**Figure 7:**
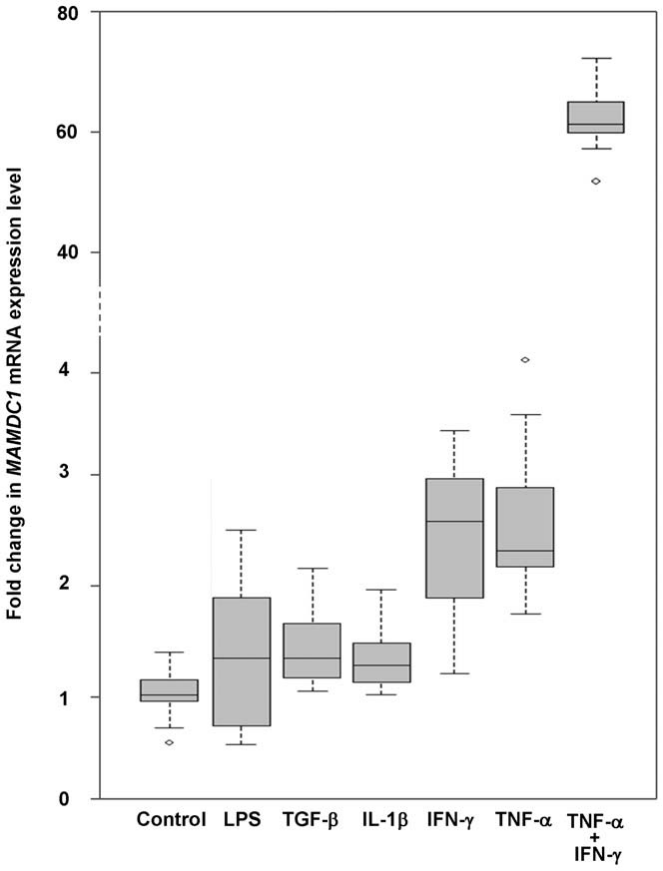
Effect of selected cytokines on MAMDC1 mRNA expression in THP-1 monocytes. THP-1 cells were treated for 24 h with LPS, TGF-β1, IL-1β, IFN-γ, TNF-α, or a combination of TNF-α and IFN-γ, and MAMDC1 mRNA expression was quantified by Real-Time PCR in triplicate experiments. The results are reported as fold changes relative to THP-1 cells grown in the absence of stimulation (control), with the smallest observation, lower quartile, median, upper quartile, and largest observation shown for each sample.

We next sought to determine whether such expression shows conditional regulation, and tested the effect of pro- and anti-inflammatory stimuli on gene transcription *in vitro*. The cell lines THP-1 (monocytic leukemia), A431 (epidermoid carcinoma), A549 (lung epithelial carcinoma), HeLa (cervix epithelial adenocarcinoma), SH-Sy5y (neuroblastoma), MCF7 (breast adenocarcinoma), HCT116 (colon carcinoma) and HEK293 (embryonic kidney cells) were first tested for *MAMDC1* mRNA expression in a quantitative qRT-PCR assay specific for the full-length isoform 1. In these experiments, moderate levels of *MAMDC1* could be detected in THP-1 and MCF7 cells, while all other cell-lines showed little or no mRNA expression (data not shown). Monocytes play a key role in inflammation and immunological diseases, and therefore we selected the THP-1 monocytic cells for the next experiments. The effect exerted on *MAMDC1* mRNA expression by TNF-α, IFN-γ and interleukin 1 beta (IL-1β), three cytokines playing a pivotal role in inflammation and in chronic inflammatory diseases such as SLE [30]–[32], by lipopolysaccharide (LPS), a potent endotoxin activating macrophage pro-inflammatory responses, and by transforming growth factor beta 1 (TGF-β1), an anti-inflammatory cytokine with pleiotropic effects in SLE and other autoimmune disorders, was then determined by qRT-PCR on THP-1 cells at 6 h and 24 h after the addition of these molecules to the culture medium. While no differences were observed 6 h post stimulation (not shown), an increase in *MAMDC1* mRNA levels was observed 24 h after the addition of either TNF-α or IFN-γ to THP-1 cells (2.5 and 2.4 fold induction, respectively, Figure 7). Of note, such increase was dramatically pronounced under the combined stimulus of these two cytokines (63 fold induction), possibly due to their known synergistic pro-inflammatory effect on gene transcription [33].

## Discussion

In the present study, we have identified association between SNPs in the novel gene *MAMDC1* and SLE in four independent samples from Finland, Sweden and the UK.

There are 2317 SNPs contained within *MAMDC1*, which covers a region of 0.8 megabases (Mb) and several linkage disequilibrium (LD) blocks (not shown). Based on the moderate effect of *MAMDC1* on SLE susceptibility, it is unlikely that this gene would appear among the top findings reported in any of the GWAs studies.

Recently, *MAMDC1* was also found associated with neuroticism in a GWA study followed by a replication in an independent sample set [34]. Four SNPs in *MAMDC1*, all located in a 3′ 37 kb region of high LD including the 10th exon, showed *P*-values of 10^−6^ to 10^−5^ in the GWA study sample and of 0.006 to 0.02 in the replication sample. However, this finding could not be supported in a follow-up association study [35]. Neuroticism is a trait that reflects a tendency toward negative mood states [36] and is linked to internalizing psychiatric conditions, such as anxiety and depression [37], [38]. With regard to SLE, this finding is of relevance since neuropsychiatric manifestations are among the ACR criteria used in the diagnosis of SLE. Furthermore, and of potential interest, exonic copy number variants in *MAMDC1* was recently shown to contribute to risk in autism spectrum disorders [39]. Unfortunately we do not have sufficient power to test for association between *MAMDC1* and different SLE neuropsychiatric manifestations.

The expression and function of the *MAMDC1* gene and protein are not well studied: besides showing expression in the rat brain and suggested to have a role in axon guidance [28], not much is known. We report here for the first time that the *MAMDC1* gene and protein were expressed in several tissues in humans, including the immune system. We could further show that *MAMDC1* mRNA is up-regulated by pro-inflammatory cytokines. Similar to previous observations made for other members of the IgCAM superfamily, such as intercellular adhesion molecule-1 (ICAM-1 [MIM 147840]) and vascular cell adhesion molecule-1 (VCAM-1 [MIM 192225]) [40]–[43], these results suggest that *MAMDC1* expression could increase during inflammation, and it is tempting to speculate that its potential role in SLE might be related to the dysregulation of immune functions typical of this disease.

Migration of leukocytes to sites of inflammation is crucial to the pathogenesis and development of inflammatory lesions in SLE and other autoimmune disorders [44]. Although the mechanisms underlying this leukocyte redistribution are still not fully understood, adhesion molecules such as those of the IgCAM superfamily have been strongly implicated in the recruitment of immune cells to sites of inflammation, and changes in their expression have been reported in rheumatoid arthritis (MIM 180300), multiple sclerosis (MIM 126200), insulin-dependent diabetes mellitus (MIM 222100), and SLE [45], [46]. Increased expression of VCAM-1 and ICAM-1 has been shown in SLE tissues such as the skin [43] and heart [40] and high levels of VCAM-1, associated with enhanced systemic TNF-α activity, was recently demonstrated to characterize SLE patients with manifest cardiovascular disease [47]. Given its putative function as an adhesion molecule, MAMDC1 might act through similar mechanisms, and it is possible that genetic alterations of its expression or function might have an impact on SLE disease predisposition and/or manifestations. Remarkably, a similar scenario has been proposed for the new SLE susceptibility gene *ITGAM* (MIM 120980), recently identified in two parallel GWA studies [12], [13]. It codes for an adhesion molecule interacting, among others, with ICAM-1 to slow down leukocyte rolling and migration to inflammatory sites [48]. Inspired by the functional similarity between *MAMDC1* and *ITGAM*, we performed a preliminary analysis of their potential interaction, by using genotyping data available (unpublished and refs [13] and [49]) for *MAMDC1* SNPs rs961616 and rs2297926 (in the entire sample set), and for *ITGAM* SNPs rs11150614 and rs11574637 (respectively in the UK sample, and in the Finnish and Swedish sample sets). A multivariate logistic regression analysis, however, did not disclose any significant interaction (data not shown).

In conclusion, we have shown here that *MAMDC1* polymorphism associates to SLE susceptibility in four sets of patients and controls from Finland, Sweden and the UK. Similar to homologous members of the IgCAM superfamily, the encoded protein has a predicted role in cellular adhesion and migration. While functional polymorphisms are yet to be identified, our data should stimulate further studies to fully appraise the role of *MAMDC1* in SLE. Moreover, genetic and/or functional analyses of its interaction(s) with novel SLE predisposing genes might hold potential for the discovery of new pathogenetic pathways.

## Materials and Methods

### Subjects and Samples

#### Ethics statement

Studies on “Identification of genes predisposing for Systemic Lupus Erythematosus (SLE)” has been approved to Professor Juha Kere by the Karolinska Institutet Research Ethics committee South at Huddinge University hospital F59 (Dnr 45/03).

#### Finnish sample sets

The original Finnish family cohort consist of 192 families (of which 86 were multiply affected by SLE), including 236 individuals affected with SLE and their healthy relatives. All SLE patients included in this cohort were interviewed by the same physician and the case records from the hospitals were reviewed [50]. All patients met the American College of Rheumatology (ACR) criteria for the diagnosis of SLE [37]. A subset of this material, including 35 multiplex families and 31 simplex families, all informative for linkage, was used for the identification and fine mapping of the 14q11.2-q23.2 locus [16], [17].

The Finnish case-control cohort consists of 86 SLE cases and 356 controls from Finland. For the collection of this material, all patients with clinical diagnosis of and SLE attending the Departments of Dermatology at Helsinki and Tampere University Central Hospitals during 1995–2005 were identified from the corresponding hospital registries, and contacted by mail or phone [51]. Unaffected unrelated family members (spouses or common-law spouses) were asked to participate in the study as control individuals, and an existing collection of unrelated individuals was also used as control. The participating patients were clinically examined by doctors working at the Department of Dermatology (SK, TH, JP) and interviewed using a structured questionnaire [51]. The diagnosis of SLE had also been verified by a rheumatologist.

#### UK sample set

The UK family cohort consists of 365 SLE parent affected trios and in this collection, diagnosis of SLE was established by telephone interview, health questionnaire and details from clinical notes [52]. All collected probands conformed to the ACR criteria for SLE [53].

#### Swedish sample set

The Swedish case-control cohort consists of 304 cases and 307 controls. All patients were interviewed and examined by a rheumatologist at the Department of Rheumatology, Karolinska University Hospital and all fulfilled four or more of the American College of Rheumatology (ACR) 1982 revised classification criteria for SLE [54]. The control samples were collected from population-based control individuals individually matched for age and sex with the patients.

The demographic and clinical characteristics of the study populations have in part been previously described [50]–[52], [54] and are reported in Table S3. All participants included in the present study gave written informed consent for participation in genetic studies on SLE and the study protocols were reviewed and approved by the local ethical committees.

### Genotyping

Selection of MS markers has been previously described [16], [17]. The SNPs were selected from dbSNP based on availability and their informativeness as of at the time the study was begun (information regarding tagging properties were limited at the time of SNP selection), with a preference for validated markers with a minor allele frequency (MAF) of >0.2. SNP positions are presented according to their location in the NCBI dbSNP Build 128.

All genotyping was performed at the Mutation Analysis Facility (MAF) at Karolinska Institutet, Huddinge, Sweden (www.maf.ki.se) using the Molecular Dynamics MegaBACE 1000 system (Global Medical Instrumentation, Albertville, MN, USA) for MS genotyping, and matrix-assisted laser desorption/ionization time-of-flight (MALDI-TOF) mass spectrometry based on allele-specific primer extension with either MassEXTEND® (hME) or iPLEX methods [55] (Sequenom Inc., San Diego, California, USA, www.sequenom.com), for SNP genotyping. PedCheck [56] was used to detect Mendelian inconsistencies, and markers deviating >10% than expected were excluded from the analysis. Hardy-Weinberg calculations were performed in controls to ensure that each marker was in equilibrium.

### Northern Blot

A pCMV6-XL4 vector containing a sequence identical to *MAMDC1* NCBI database entry AY369208.1 was purchased from OriGene Technologies (Rockville, MD, USA) and entirely sequenced, identifying an additional 108 bp of 5′ untranslated region (UTR) from *MAMDC1* exon 1a, and a G to T nucleotide change in exon 9 (corresponding to the SNP rs12590500). The *MAMDC1* full-length cDNA was excised from the vector using Not 1 restriction digesion (New England Biolabs, Ipswich, MA, USA) and gel purified. Fifty nano grams (ng) of purified cDNA were then labelled with P^32^-dCTP (GE Healthcare, Buckinghamshire, UK) by random priming and was used to probe two human multiple tissue polyA+ RNA Northern blots (HB2010 and HB2011, OriGene Technologies) according to manufacturer's instructions. Twenty five ng of β-Actin cDNA control probe were used for normalization (OriGene Technologies). Exposure to Hyperfilm MP (GE Healthcare) was done for three days.

### Immunohistochemistry

Formalin-fixed paraffin-embedded tissue sections from testis (n = 2), kidney (n = 1), duodenum (n = 2), placenta (n = 2), cutaneous squamous cell carcinoma (n = 4), and SLE skin (n = 9), were obtained from the Departments of Pathology and Dermatopathology, Helsinki University Central Hospital, Finland. The SLE diagnoses were based on clinical and laboratory data (SK), and confirmed histologically by an experienced dermatopathologist. The use of archived paraffin-embedded material was approved by the corresponding Ethical Review Board of the Helsinki University Central Hospital, Finland.

IHC analysis was performed using the peroxidase-conjugated EnVision+ peroxidase technique (Dual Link System, Peroxidase, DakoCytomation, Glostrup, Denmark), with diaminobenzidine (DAB) as chromogenic substrate and Mayer hematoxylin as counterstain. Incubation with primary rabbit polyclonal antibody (1∶20, HPA003084, Atlas Antibodies, Stockholm, Sweden), in PBS containing 1% bovine serum albumin (BSA, Sigma-Aldrich), was performed for 30 min at room temperature. Rabbit immunoglobulin G serum (1∶20, Zymed Laboratories Inc., South San Francisco, CA, USA) or 1% BSA in PBS was used as a negative control.

Immunohistochemical specimens were analyzed by three different investigators (TMJ, US-K, A R-S) under a light microscope at 200× magnification. Staining of 10 or more cells was interpreted as positive result.

### THP-1 Cell Stimulations

THP-1 monocytes were plated on 6-well-plates (1.4×10^6^ cells/well) and grown overnight in RPMI 1640 medium (GIBCO Invitrogen Life Technologies, Paisley, Scotland) supplemented with 10% FCS, 1 mM sodium pyruvate, 10 mM HEPES, 100 U of penicillin, 100 µg/ml streptomycin and 0.05 mM β-mercaptoethanol. The cells were then treated with 1µg/ml LPS (Sigma, St. Louis, MO, USA), 10 ng/ml TGF-β1 (Sigma), 5 U/ml IL-1β (Roche Molecular Biochemicals, Indiananpolis, IN, USA), 10 ng/ml IFN-γ (Sigma), 50 ng/ml TNF-α (Sigma), or a combination of TNF-α and IFN-γ. Stimulation was allowed to proceed for 6 and 24 h. All experiments were carried out in triplicate and cells grown in normal medium were used as controls.

### Quantitative qRT-PCR

Total RNA was extracted from lysed cells using the RNeasy Mini-kit (QIAGEN Inc, Hilden, Germany) and reverse transcribed to cDNA using SuperScript™ III Reverse Transcriptase reagents (Invitrogen, Carlsbad, CA, USA), according to manufacturers' instructions.

Primers specific to the *MAMDC1* full-length isoform 1 (forward: 5′-GATCTCTGGCCAAGGAGTGT-3′; reverse: 5′-GCCTGAGTGCACAATACGAA-3′) were designed with the Primer Express 2.0 software (Applied Biosystems, Foster City, CA, USA). Quantitative qRT-PCR reactions, with cDNA as template, were performed in triplicates with the 7500 Fast Real-Time PCR system using SYBR green chemistry and standard protocols (Applied Biosystems).

After normalization to the endogenous housekeeping gene *GAPDH*, *MAMDC1* level of expression in each sample was determined by the comparative C_T_ method of relative quantification, and expressed in arbitrary units relative to a randomly chosen reference sample or to unstimulated cells.

### Statistics

The disease association was initially mined by Haplotype Pattern Mining (HPM, 50,000 permutations) [22] and Pedigree Disequilibrium test (PDT) [21]. HPM is a method based on discovering recurrent marker patterns and has been shown to be robust and powerful for sparse marker maps. PDT integrates extended families information into the more traditional Transmission Disequilibrium Test.

Single marker association for the fine mapping stage was analyzed using two different methods; PDTPHASE in the family cohorts and COCAPHASE in the case control cohorts [21].

Meta-analysis of the case control and family data was performed using the Kazeem and Farrell [57] fixed effect model implemented in the R package catmap1.5 [www.r-project.org]. Heterogeneity was assessed using a standard Q- test.

To take into account multiple testing in the fine mapping step, the nominal significance threshold of *P* = 0.05 was corrected by finding the number of independent SNPs, using a Principal Component Analysis of the SNPs correlation matrix [58]. Out of the 24 markers genotyped in this study, 16 resulted to be statistically independent, which fixed the significance threshold to *P* = 0.0032.

Haplotypes were tested using “haplo.stats 1.3.0” software from R. Here, haplotype inference is performed with a standard Expectation Maximization method and the association is tested with a Generalized Linear Model, which uses haplotypes posterior probabilities as weights. Haplotypes were tested over blocks of consecutive markers as defined in Haploview 4.1 (http://www.broad.mit.edu/mpg/haploview) [59].

### Web Resources

NCBI (http://www.ncbi.nlm.nih.gov/)

dbSNP (http://www.ncbi.nlm.nih.gov/SNP/)

Online Mendelian Inheritance in Man (OMIM) (http://www.ncbi.nih.gov/entrez/query.fcgi?db=OMIM)

Primer3 (http://frodo.wi.mit.edu/primer3/primer3_code.html)

SignalP 3.0 (http://www.cbs.dtu.dk/services/SignalP/)

Swedish Human Protein Atlas program (www.proteinatals.org)

Mutation Analysis Facility (MAF) at Karolinska Institutet, Stockholm, Sweden (www.maf.ki.se)

Sequenom Inc. (www.sequenom.com)

Haploview 4.1 (http://www.broad.mit.edu/mpg/haploview)

R (www.r-project.org)

ClustalW2 (http://www.ebi.ac.uk/Tools/clustalw2/index.html)

## Supporting Information

Table S1 Markers genotyped in the study
(0.14 MB DOC)

Table S2 P-values and ORs for the combined analysis
(0.05 MB DOC)

Table S3 Demographic and clinical characteristics of the study populations, as defined by the revised ACR criteria for SLE
(0.04 MB DOC)
