# Supplementary material for: Identification of MAMDC1 as a Candidate Susceptibility Gene for Systemic Lupus Erythematosus (SLE)
Source: PLoS One. 2009 Dec 7;4(12):e8037. doi: 10.1371/journal.pone.0008037 (PMC2785483; doi:10.1371/journal.pone.0008037)
Supplement: Table S1 — Markers genotyped in the study (0.14 MB DOC) [file pone.0008037.s001.doc]

**Table S1.** Markers genotyped in the study (continued on next page).

| **Marker name** | **Marker location** | **Genotype** | **MAF**  **FIN family material** | **MAF**  **UK family material** | **MAF**  **FIN case/**  **control**  **material** | **MAF**  **SWE case/**  **control**  **material** |
| --- | --- | --- | --- | --- | --- | --- |
| *D14S69* | *36129029-36129239* |  |  |  |  |  |
| *D14S1432* | *37650816-37650966* |  |  |  |  |  |
| *D14S1428* | *38486568-38486748* |  |  |  |  |  |
| *D14S1039* | *39354221-39354372* |  |  |  |  |  |
| *D14S579* | *39995255-39995558* |  |  |  |  |  |
| *D14S301* | *42004368-42004648* |  |  |  |  |  |
| *D14S1068* | *46203056-46203268* |  |  |  |  |  |
| rs961616 | 46591239 | A/G | 0.412 (G) | 0.355 (G) | 0.357 (G) | 0.394 (G) |
| rs8009302 | 46598220 | A/G | 0.082 (A) | 0.075 (A) | 0.064 (A) | 0.089 (A) |
| rs1547809 | 46602004 | A/G | 0.167 (G) |  |  |  |
| rs1952220 | 46690298 | C/T | 0.188 (C) | 0.155 (C) | 0.164 (C) | 0.174 (C) |
| *D14S541* | *46696465-46696632* |  |  |  |  |  |
| rs10132207 | 46730126 | A/G | 0.3 (G) | 0.39 (G) | 0.367 (G) | 0.352 (G) |
| rs1958087 | 46746037 | A/G | 0.158 (A) | 0.141 (A) | 0.137 (A) | 0.149 (A) |
| rs8012244 | 46778036 | A/T | 0.475 (A) | 0.496 (A) | 0.463 (A) | 0.497 (A) |
| rs10484192 | 46778147 | A/G | 0.111 (A) | 0.069 (A) | 0.118 (A) | 0.087 (A) |
| rs10484188 | 46801979 | A/G | 0.491 (G) | 0.403 (A) | 0.477 (A) | 0.396 (A) |
| rs1571105 | 46804889 | C/T | 0.213 (C) | 0.192 (C) | 0.199 (C) | 0.174 (C) |
| rs2148509 | 46806044 | C/T | 0.213 (C) | 0.192 (C) | 0.199 (C) | 0.179 (C) |
| rs1952213 | 46808021 | C/G | 0.295 (C) | 0.355 (C) | 0.278 (C) | 0.332 (C) |
| rs2148510 | 46808221 | C/T | 0.218 (T) | 0.189 (T) | 0.198 (T) | 0.171 (T) |
| rs1571106 | 46812303 | A/C | 0.114 (A) | 0.109 (A) | 0.101 (A) | 0.109 (A) |
| rs4900734 | 46814157 | C/T | 0.32 (T) | 0.288 (T) | 0.313 (T) | 0.272 (T) |
| rs2899998 | 46822913 | A/G | 0.142 (A) | 0.191 (A) | 0.133 (A) | 0.182 (A) |
| rs9671434 | 46826017 | G/T | 0.193 (G) | 0.101 (G) | 0.189 (G) | 0.125 (G) |
| rs10144408 | 46834346 | C/T | 0.071 (C) | 0.166 (C) | 0.074 (C) | 0.155 (C) |
| rs4900736 | 46838439 | A/C | 0.219 (C) | 0.193 (C) | 0.196 (C) | 0.180 (C) |
| rs12433300 | 46843996 | A/T | 0.330 (T) | 0.296 (T) | 0.316 (T) | 0.278 (T) |
| rs2297926 | 46844251 | A/C | 0.075 (A) | 0.178 (A) | 0.084 (A) | 0.163 (A) |
| rs9323131 | 46868216 | A/C | 0.084 (C) | 0.165 (C) | 0.084 (C) | 0.115 (C) |
| rs1815638 | 46871007 | C/T | 0.203 (T) | 0.129 (T) | 0.201 (T) | 0.160 (T) |
| rs2277472 | 46881821 | C/T | 0.187 (T) | 0.094 (T) | 0.193 (T) | 0.134 (T) |
| rs2068986 | 46939119 | G/T | 0.239 (T) | 0.209 (T) | 0.241 (T) | 0.214 (T) |
| rs1955810 | 47075551 | A/G | 0.32 (G) |  |  |  |
| *D14S976* | *47157985-47158156* |  |  |  |  |  |
| rs1958628 | 47177390 | A/G | 0.341 (G) |  |  |  |
| *GATA85A11* | *48162058-48162420* |  |  |  |  |  |
| *D14S1031* | *49381035-49381276* |  |  |  |  |  |
| rs2356895 | 50898162 | A/G | 0.208 (G) |  |  |  |
| rs999906 | 51269968 | A/T | 0.45 (A) |  |  |  |
| rs1953866 | 51387587 | A/G | 0.247 (A) |  |  |  |
| rs946615 | 51551667 | C/T | 0.264 (T) |  |  |  |
| *D14S1018* | *51563972-51564176* |  |  |  |  |  |
| rs1898475 | 51735245 | C/T | 0.432 (T) |  |  |  |
| rs708506 | 51868703 | G/T | 0.371 (G) |  |  |  |
| rs708534 | 51935900 | A/G | 0.382 (A) |  |  |  |
| rs3742533 | 52051151 | G/T | 0.471 (T) |  |  |  |
| rs762063 | 52168652 | A/G | 0.461 (G) |  |  |  |
| rs912861 | 52403040 | C/G | 0.106 (G) |  |  |  |
| D14S989 | 52774794-52774972 |  |  |  |  |  |
| *rs763328* | *52776759* | *A/G* | *0.045 (G)* |  |  |  |
| rs918935 | 52829512 | A/G | 0.486 (G) |  |  |  |
| rs735199 | 52884983 | A/G | 0.49 (A) |  |  |  |
| *D14S139* | *53039214-53039459* |  |  |  |  |  |
| rs2077940 | 53061455 | A/G | 0.382 (G) |  |  |  |
| D14S747 | 53637396-53637596 |  |  |  |  |  |
| *D14S991* | *54293775-54293933* |  |  |  |  |  |
| *D14S276* | *54752769-54752873* |  |  |  |  |  |
| *GATA84B02* | *55621435-55621550* |  |  |  |  |  |
| *D14S66* | *56120401-56120590* |  |  |  |  |  |

FM1 = fine mapping step 1, FM2 = fine mapping step 2 (see manuscript for details).

MAF = minor allele frequency.
