# Supplementary material for: Identification of MAMDC1 as a Candidate Susceptibility Gene for Systemic Lupus Erythematosus (SLE)
Source: PLoS One. 2009 Dec 7;4(12):e8037. doi: 10.1371/journal.pone.0008037 (PMC2785483; doi:10.1371/journal.pone.0008037)
Supplement: Table S2 — P-values and ORs for the combined analysis (0.05 MB DOC) [file pone.0008037.s002.doc]

**Table S2.** *P*-values and ORs for the combined analysis.

| **SNP ID** | ***P*-value** | **OR** | **OR (95% CI)** |
| --- | --- | --- | --- |
| rs961616 | 0.001 | 1.293 | 1.103 - 1.513 |
| rs8009302 | 0.166 |  |  |
| rs1952220 | 0.358 |  |  |
| rs10132207 | 0.040 |  |  |
| rs1958087 | 0.410 |  |  |
| rs8012244 | 0.315 |  |  |
| rs10484192 | 0.306 |  |  |
| rs10484188 | 0.816 |  |  |
| rs1571105 | 0.811 |  |  |
| rs2148509 | 0.814 |  |  |
| rs1952213 | 0.792 |  |  |
| rs2148510 | 0.658 |  |  |
| rs1571106 | 0.311 |  |  |
| rs4900734 | 0.542 |  |  |
| rs2899998 | 0.751 |  |  |
| rs9671434 | 0.480 |  |  |
| rs10144408 | 0.297 |  |  |
| rs4900736 | 0.637 |  |  |
| rs12433300 | 0.415 |  |  |
| rs2297926 | 0.003 | 1.349 | 1.109 – 1.640 |
| rs9323131 | 0.926 |  |  |
| rs1815638 | 0.710 |  |  |
| rs2277472 | 0.157 |  |  |
| rs2068986 | 0.910 |  |  |
