# Supplementary material for: Identification of MAMDC1 as a Candidate Susceptibility Gene for Systemic Lupus Erythematosus (SLE)
Source: PLoS One. 2009 Dec 7;4(12):e8037. doi: 10.1371/journal.pone.0008037 (PMC2785483; doi:10.1371/journal.pone.0008037)
Supplement: Table S3 — Demographic and clinical characteristics of the study populations, as defined by the revised ACR criteria for SLE (0.04 MB DOC) [file pone.0008037.s003.doc]

**Table S3.** Demographic and clinical characteristics of the study populations, as defined by the revised ACR criteria for SLE [1].

|  | **Finnish**  **Family**  **material (n=236)** | **British**  **Family**  **material (n=366)** | **Finnish**  **Case/control**  **Material**  **(n=90)** | **Swedish**  **Case/control**  **Material**  **(n=304)** |
| --- | --- | --- | --- | --- |
| Females | 94% | 91% | 93% | 90% |
| Mean age at onset (range) | 29 (1–66) | 24 (3-45) | 31 (8-73) | n.a |
| Mean age at diagnosis (range) | 33 (6–72) | 27 (10-47) | 35 (13-76) | 31 (7-74) |
| Butterfly rash | 51% | 82% | 74% | 52% |
| Discoid rash | 10% |  | 41% | 17% |
| Photo- sensitivity | 69% | 68% | 80% | 52% |
| Mouth ulcers | 18% | 67% | 16% | 34% |
| Arthritis | 83% | 72% | 64% | 87% |
| Pleuritis | 18% | 27% | n.a. | 40% |
| Pericarditis | 16% |  | n.a. | 19% |
| Nephritis | 30% | 36% | 20% | 41% |
| Leukopenia | 68% | n.a. | 37% | 50% |
| Thrombocytopenia | 16% | 24% | 16% | 21% |

NOTE. All values are presented as % over available values.

n.a.= not available

**References**

1. Tan EM, Cohen AS, Fries JF, Masi AT, McShane DJ, et al. (1982) The 1982 revised criteria for the classification of systemic lupus erythematosus. Arthritis Rheum 25: 1271-1277.
